# Supplementary material for: A Humanized Yeast Phenomic Model of Deoxycytidine Kinase to Predict Genetic Buffering of Nucleoside Analog Cytotoxicity
Source: Genes (Basel). 2019 Sep 30;10(10):770. doi: 10.3390/genes10100770 (PMC6826991; doi:10.3390/genes10100770)
Supplement: Supplementary file 1 [file genes-10-00770-s001.zip › Additional_Files/Additional_File7_GOTermHeatmaps.zip!/Additional_File7_GOTermHeatmaps/mRNA_processing.pdf]

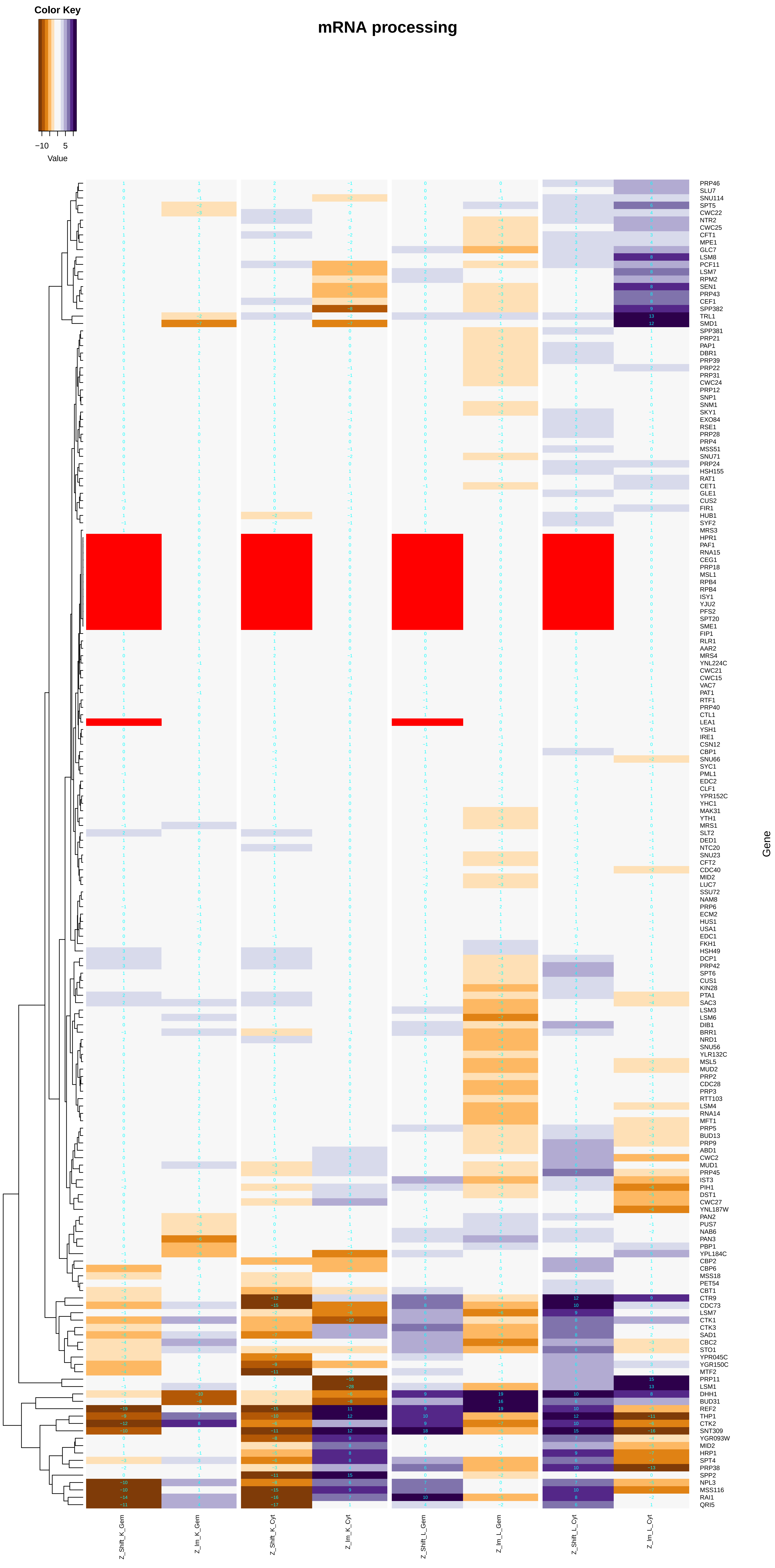
Color Key
−10
5
Value
mRNA processing
Gene
Z_Shift_K_Gem
Z_Im_K_Gem
Z_Shift_K_Cyt
Z_Im_K_Cyt
Z_Shift_L_Gem
Z_Im_L_Gem
Z_Shift_L_Cyt
Z_Im_L_Cyt
PRP46 SLU7 SNU114 SPT5 CWC22 NTR2 CWC25 CFT1 MPE1 GLC7 LSM8 PCF11 LSM7 RPM2 SEN1 PRP43 CEF1 SPP382 TRL1 SMD1 SPP381 PRP21 PAP1 DBR1 PRP39 PRP22 PRP31 CWC24 PRP12 SNP1 SNM1 SKY1 EXO84 RSE1 PRP28 PRP4 MSS51 SNU71 PRP24 HSH155 RAT1 CET1 GLE1 CUS2 FIR1 HUB1 SYF2 MRS3 HPR1 PAF1 RNA15 CEG1 PRP18 MSL1 RPB4 RPB4 ISY1 YJU2 PFS2 SPT20 SME1 FIP1 RLR1 AAR2 MRS4 YNL224C CWC21 CWC15 VAC7 PAT1 RTF1 PRP40 CTL1 LEA1 YSH1 IRE1 CSN12 CBP1 SNU66 SYC1 PML1 EDC2 CLF1 YPR152C YHC1 MAK31 YTH1 MRS1 SLT2 DED1 NTC20 SNU23 CFT2 CDC40 MID2 LUC7 SSU72 NAM8 PRP6 ECM2 HUS1 USA1 EDC1 FKH1 HSH49 DCP1 PRP42 SPT6 CUS1 KIN28 PTA1 SAC3 LSM3 LSM6 DIB1 BRR1 NRD1 SNU56 YLR132C MSL5 MUD2 PRP2 CDC28 PRP3 RTT103 LSM4 RNA14 MFT1 PRP5 BUD13 PRP9 ABD1 CWC2 MUD1 PRP45 IST3 PIH1 DST1 CWC27 YNL187W PAN2 PUS7 NAB6 PAN3 PBP1 YPL184C CBP2 CBP6 MSS18 PET54 CBT1 CTR9 CDC73 LSM7 CTK1 CTK3 SAD1 CBC2 STO1 YPR045C YGR150C MTF2 PRP11 LSM1 DHH1 BUD31 REF2 THP1 CTK2 SNT309 YGR093W MID2 HRP1 SPT4 PRP38 SPP2 NPL3 MSS116 RAI1 QRI5

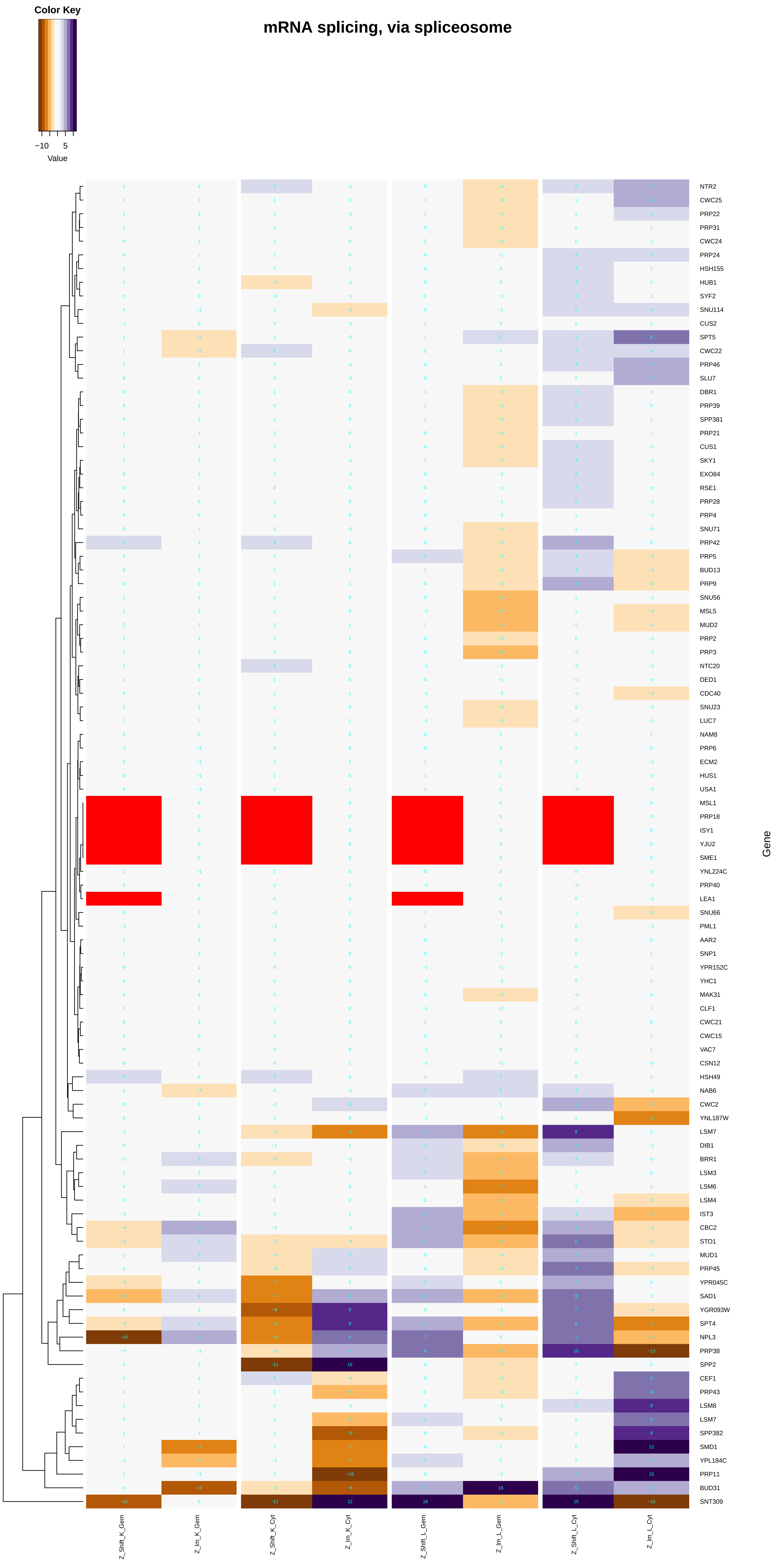

Color Key
−10
5
Value
mRNA splicing, via spliceosome
Gene
Z_Shift_K_Gem
Z_Im_K_Gem
Z_Shift_K_Cyt
Z_Im_K_Cyt
Z_Shift_L_Gem
Z_Im_L_Gem
Z_Shift_L_Cyt
Z_Im_L_Cyt
NTR2
CWC25
PRP22
PRP31
CWC24
PRP24
HSH155
HUB1
SYF2
SNU114
CUS2
SPT5
CWC22
PRP46
SLU7
DBR1
PRP39
SPP381
PRP21
CUS1
SKY1
EXO84
RSE1
PRP28
PRP4
SNU71
PRP42
PRP5
BUD13
PRP9
SNU56
MSL5
MUD2
PRP2
PRP3
NTC20
DED1
CDC40
SNU23
LUC7
NAM8
PRP6
ECM2
HUS1
USA1
MSL1
PRP18
ISY1
YJU2
SME1
YNL224C
PRP40
LEA1
SNU66
PML1
AAR2
SNP1
YPR152C
YHC1
MAK31
CLF1
CWC21
CWC15
VAC7
CSN12
HSH49
NAB6
CWC2
YNL187W
LSM7
DIB1
BRR1
LSM3
LSM6
LSM4
IST3
CBC2
STO1
MUD1
PRP45
YPR045C
SAD1
YGR093W
SPT4
NPL3
PRP38
SPP2
CEF1
PRP43
LSM8
LSM7
SPP382
SMD1
YPL184C
PRP11
BUD31
SNT309

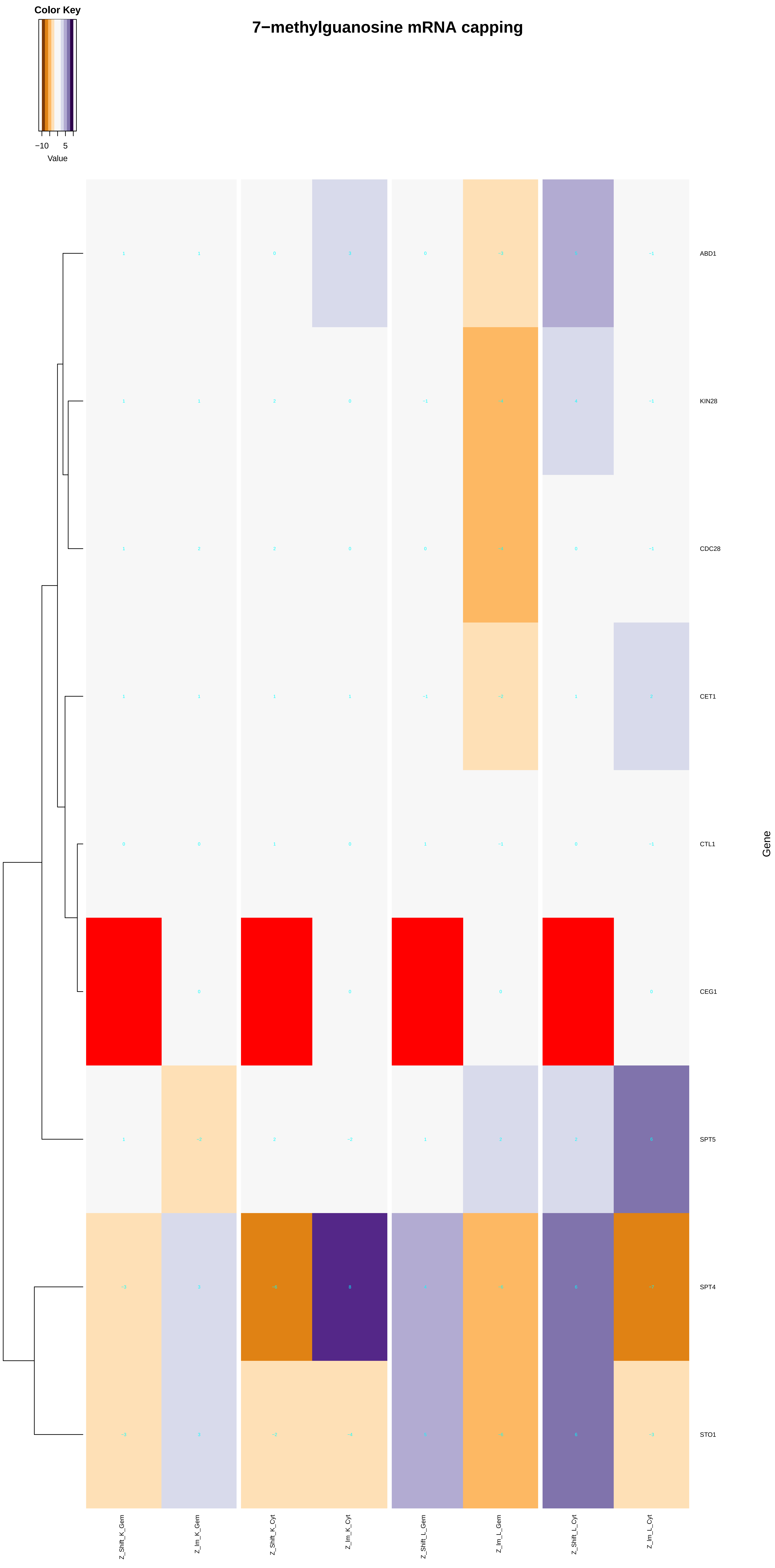
7−methylguanosine mRNA capping
Color Key
−10
5
Value
ABD1
KIN28
CDC28
CET1
CTL1
CEG1
SPT5
SPT4
STO1
Gene
Z_Shift_K_Gem
Z_Im_K_Gem
Z_Shift_K_Cyt
Z_Im_K_Cyt
Z_Shift_L_Gem
Z_Im_L_Gem
Z_Shift_L_Cyt
Z_Im_L_Cyt

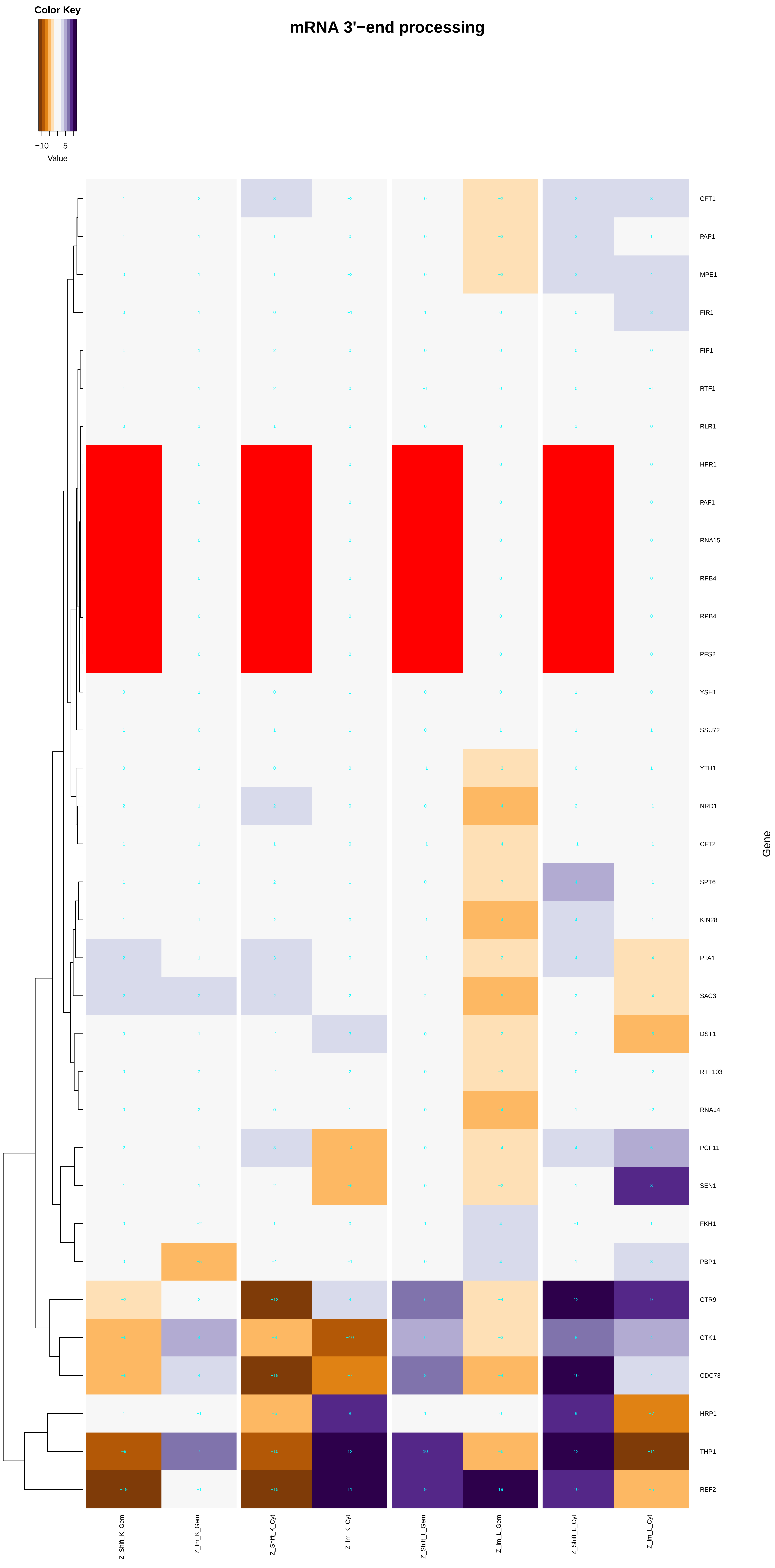

Color Key
−10
5
Value
mRNA 3'−end processing
CFT1
PAP1
MPE1
FIR1
FIP1
RTF1
RLR1
HPR1
PAF1
RNA15
RPB4
RPB4
PFS2
YSH1
SSU72
YTH1
NRD1
CFT2
SPT6
KIN28
PTA1
SAC3
DST1
RTT103
RNA14
PCF11
SEN1
FKH1
PBP1
CTR9
CTK1
CDC73
HRP1
THP1
REF2
Gene
Z_Shift_K_Gem
Z_Im_K_Gem
Z_Shift_K_Cyt
Z_Im_K_Cyt
Z_Shift_L_Gem
Z_Im_L_Gem
Z_Shift_L_Cyt
Z_Im_L_Cyt

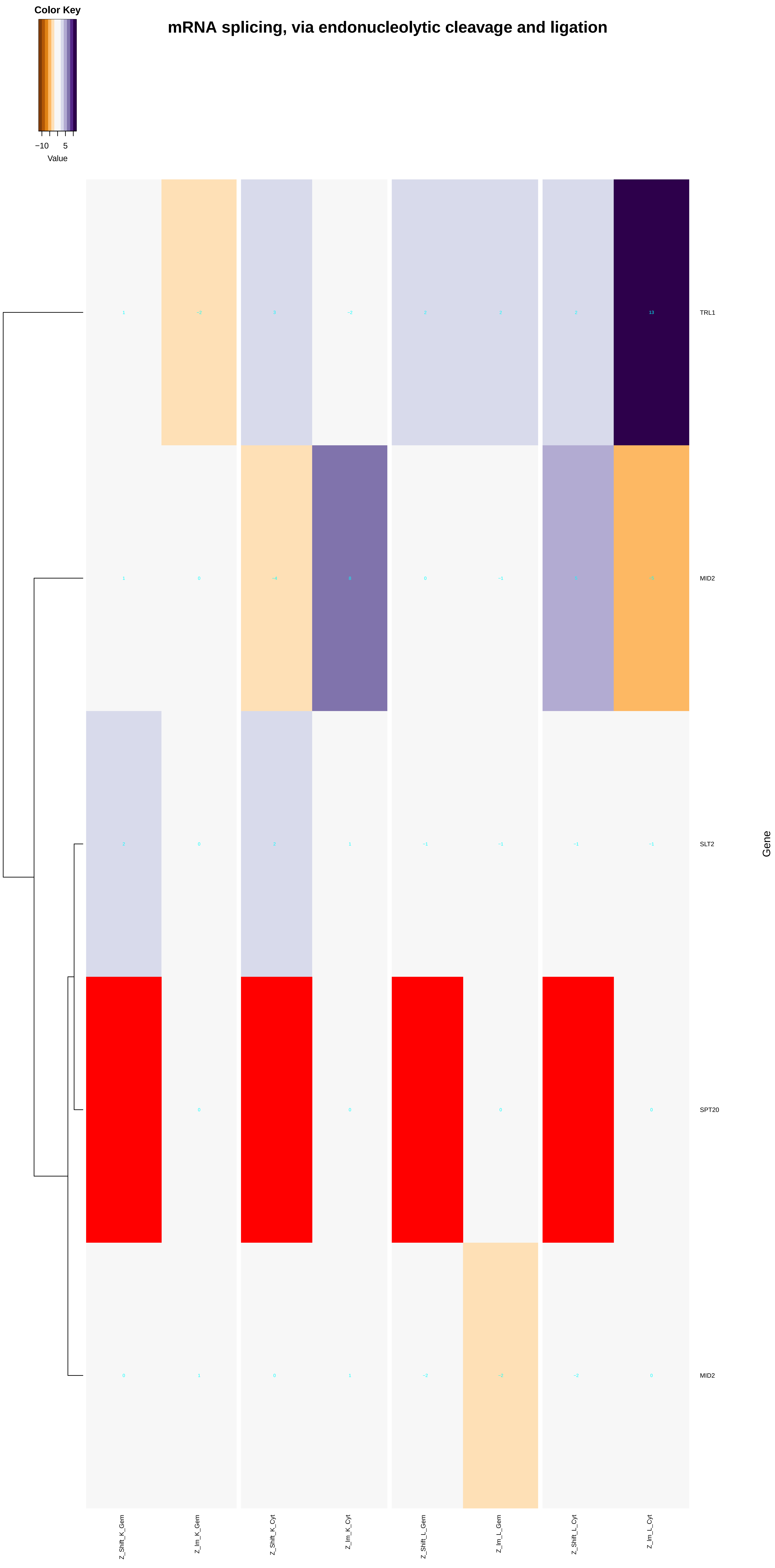
mRNA splicing, via endonucleolytic cleavage and ligation
Color Key
−10
5
Value
TRL1
MID2
SLT2
SPT20
MID2
Gene
Z_Shift_K_Gem
Z_Im_K_Gem
Z_Shift_K_Cyt
Z_Im_K_Cyt
Z_Shift_L_Gem
Z_Im_L_Gem
Z_Shift_L_Cyt
Z_Im_L_Cyt

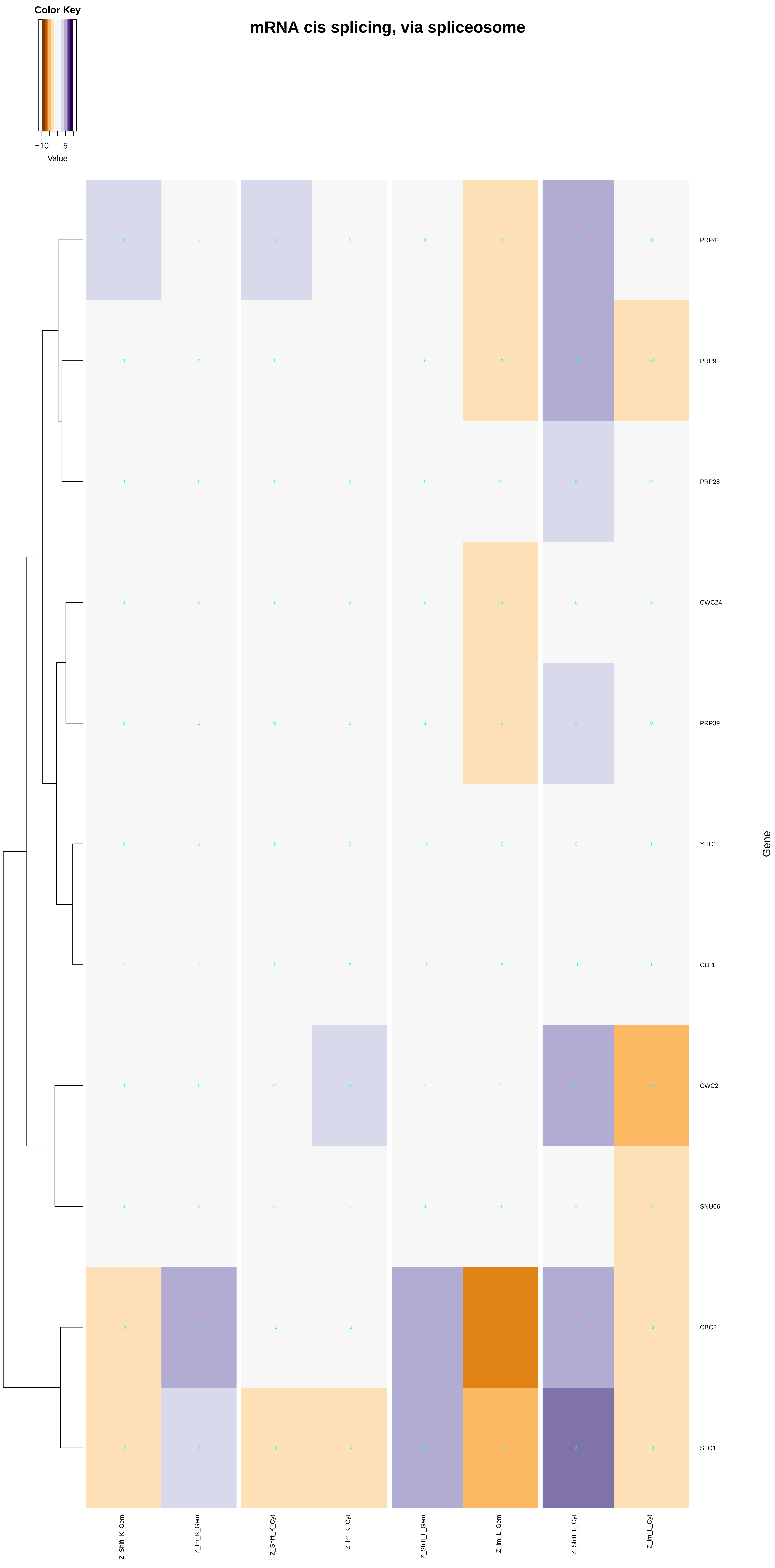
Color Key
−10
5
Value
mRNA cis splicing, via spliceosome
PRP42
PRP9
PRP28
CWC24
PRP39
YHC1
CLF1
CWC2
SNU66
CBC2
STO1
Gene
Z_Shift_K_Gem
Z_Im_K_Gem
Z_Shift_K_Cyt
Z_Im_K_Cyt
Z_Shift_L_Gem
Z_Im_L_Gem
Z_Shift_L_Cyt
Z_Im_L_Cyt

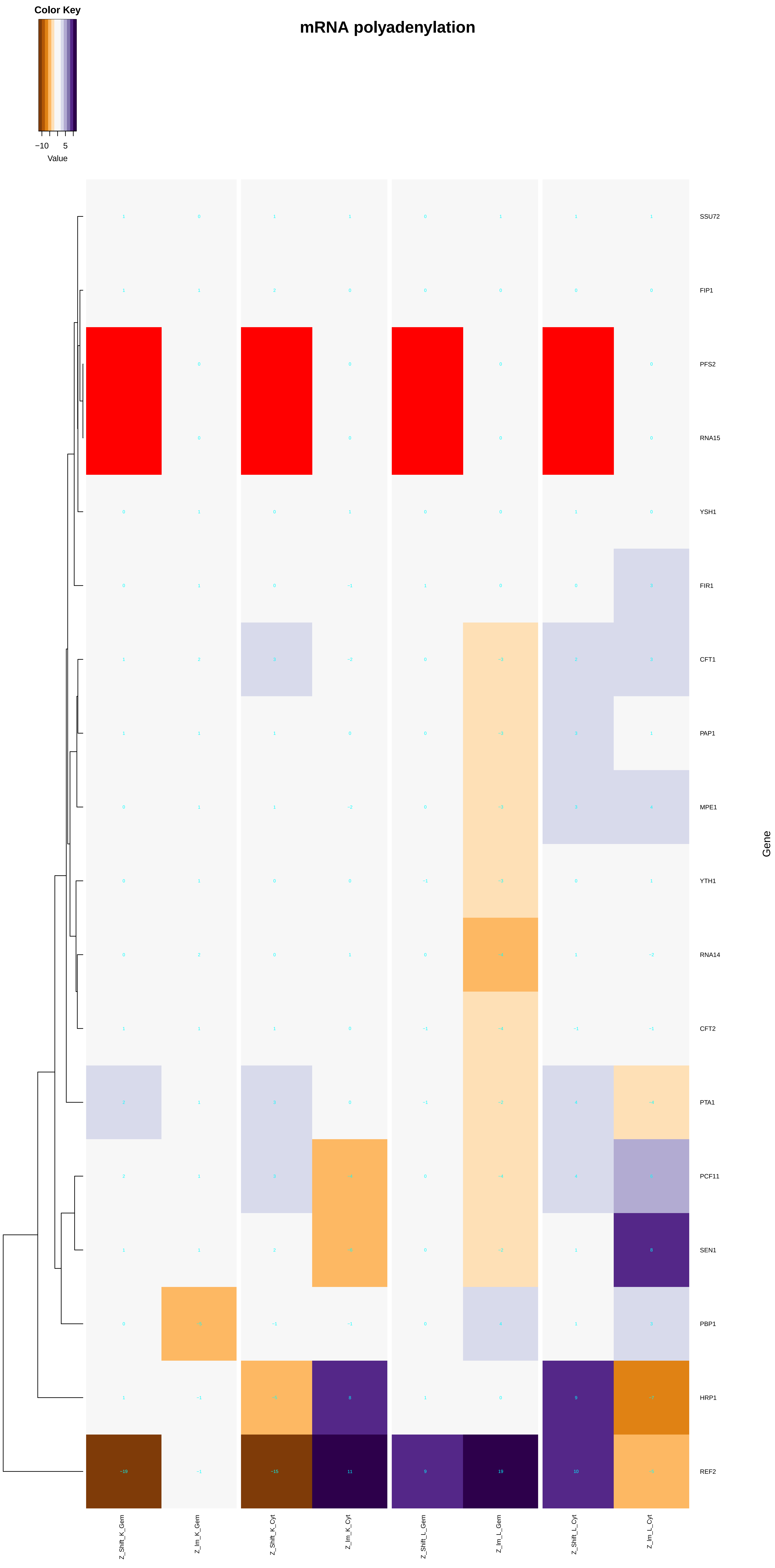
mRNA polyadenylation
Color Key
−10
5
Value
SSU72
FIP1
PFS2
RNA15
YSH1
FIR1
CFT1
PAP1
MPE1
YTH1
RNA14
CFT2
PTA1
PCF11
SEN1
PBP1
HRP1
REF2
Gene
Z_Shift_K_Gem
Z_Im_K_Gem
Z_Shift_K_Cyt
Z_Im_K_Cyt
Z_Shift_L_Gem
Z_Im_L_Gem
Z_Shift_L_Cyt
Z_Im_L_Cyt

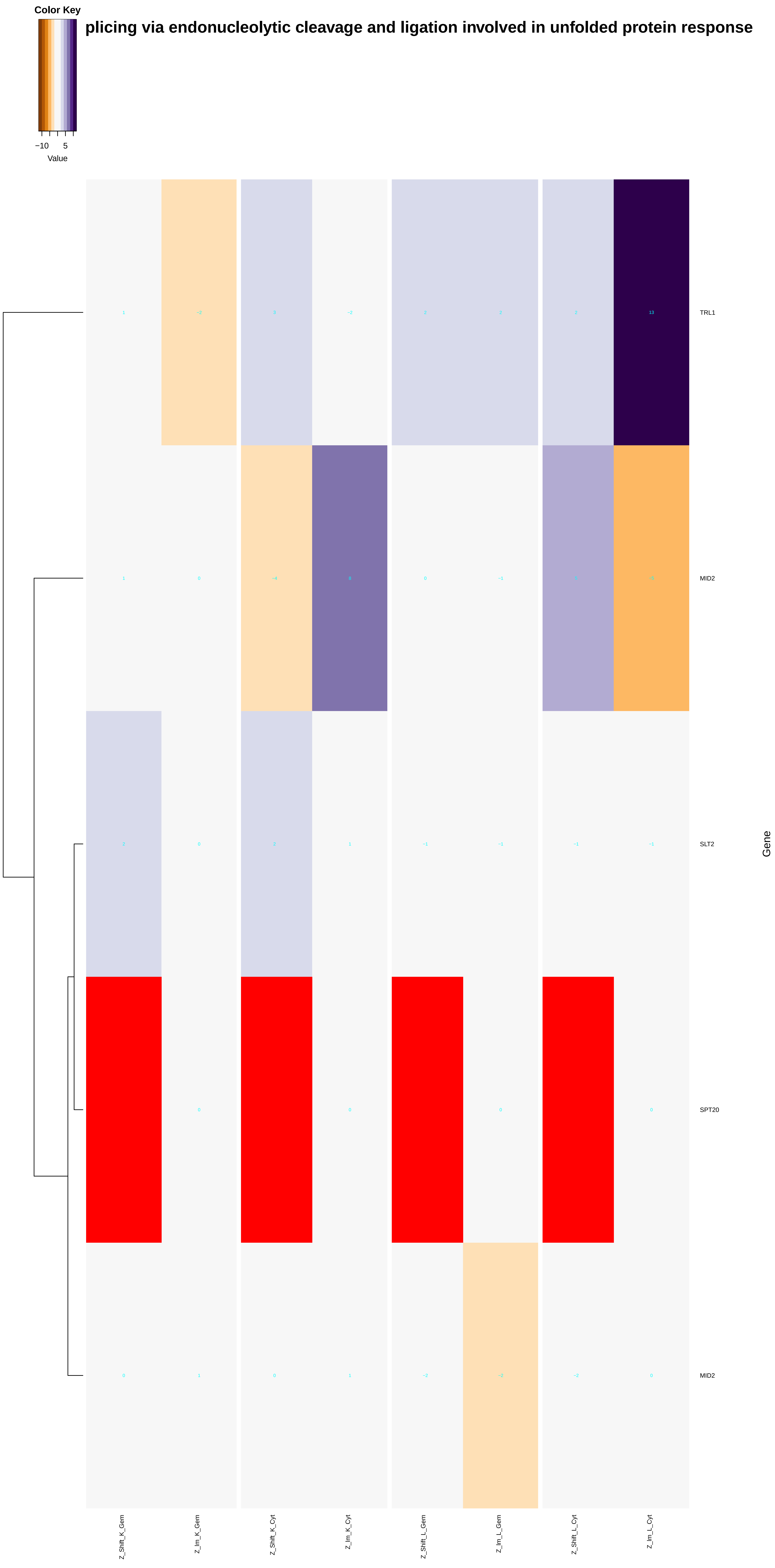

plicing via endonucleolytic cleavage and ligation involved in unfolded protein response
Color Key
−10
5
Value
TRL1
MID2
SLT2
SPT20
MID2
Gene
Z_Shift_K_Gem
Z_Im_K_Gem
Z_Shift_K_Cyt
Z_Im_K_Cyt
Z_Shift_L_Gem
Z_Im_L_Gem
Z_Shift_L_Cyt
Z_Im_L_Cyt
